# Exploring the Impact of the Multimodal CAPABLE eHealth Intervention on Health-Related Quality of Life in Patients With Melanoma Undergoing Immune-Checkpoint Inhibition: Prospective Pilot Study

**DOI:** 10.2196/58938

**Published:** 2025-01-30

**Authors:** Itske Fraterman, Lucia Sacchi, Henk Mallo, Valentina Tibollo, Savannah Lucia Catherina Glaser, Stephanie Medlock, Ronald Cornet, Matteo Gabetta, Vitali Hisko, Vadzim Khadakou, Ella Barkan, Laura Del Campo, David Glasspool, Alexandra Kogan, Giordano Lanzola, Roy Leizer, Manuel Ottaviano, Mor Peleg, Konrad Śniatała, Aneta Lisowska, Szymon Wilk, Enea Parimbelli, Silvana Quaglini, Mimma Rizzo, Laura Deborah Locati, Annelies Boekhout, Lonneke V van de Poll-Franse, Sofie Wilgenhof

**Affiliations:** 1 Department of Psychosocial Research and Epidemiology, The Netherlands Cancer Institute, Plesmanlaan 121, Amsterdam, 1066CX, Netherlands, 31 0621885919; 2 Department of Electric, Computer and Biomedical Engineering, University of Pavia, Pavia, Italy; 3 Department of Medical Oncology, Antoni van Leeuwenhoek, Amsterdam, Netherlands; 4 Laboratory of Informatics and Systems Engineering for Clinical Research, Istituti Clinici Scientifici Maugeri SpA SB IRCCS, Pavia, Italy; 5 Medical Informatics, Amsterdam UMC - University of Amsterdam, Amsterdam, Netherlands; 6 Methodology and Digital Health, Amsterdam Public Health, Amsterdam, Netherlands; 7 BITSENS JSC, Vilnius, Lithuania; 8 Department of Artificial Intelligence for Accelerated Healthcare and Life Sciences Discovery, IBM Research, University of Haifa, Haifa, Israel; 9 Associazione Italiana Malati di Cancro, Rome, Italy; 10 Deontics Ltd, London, United Kingdom; 11 Department of Information Systems, University of Haifa, Haifa, Israel; 12 Life Supporting Technologies, Universidad Politécnica de Madrid, Madrid, Spain; 13 Institute of Computing Science, Poznan University of Technology, Poznan, Poland; 14 Division of Medical Oncology, Azienda Ospedaliero Universitaria Consorziale Policlinico di Bari, Bari, Italy; 15 Department of Internal Medicine and Medical Therapy, University of Pavia, Pavia, Italy; 16 Medical Oncology Unit, Istituti Clinici Scientifici Maugeri IRCCS, Pavia, Italy; 17 Department of Medical and Clinical Psychology, Center of Research on Psychological and Somatic Disorders (CoRPS), Tilburg University, Tilburg, Netherlands

**Keywords:** eHealth, melanoma, cancer, fatigue, quality of life, intervention, pilot study, exploratory, health-related, interventions, symptom, monitoring, well-being, immunotherapy, immune-related, immune-checkpoint inhibitor, patient, feasibility, smartphone, app, smartwatch, linear regression model, mobile phone

## Abstract

**Background:**

Patients with melanoma receiving immunotherapy with immune-checkpoint inhibitors often experience immune-related adverse events, cancer-related fatigue, and emotional distress, affecting health-related quality of life (HRQoL) and clinical outcome to immunotherapy. eHealth tools can aid patients with cancer in addressing issues, such as adverse events and psychosocial well-being, from various perspectives.

**Objective:**

This study aimed to explore the effect of the Cancer Patients Better Life Experience (CAPABLE) system, accessed through a mobile app, on HRQoL compared with a matched historical control group receiving standard care. CAPABLE is an extensively tested eHealth app, including educational material, remote symptom monitoring, and well-being interventions.

**Methods:**

This prospective pilot study compared an exploratory cohort that received the CAPABLE smartphone app and a multisensory smartwatch for 6 months (intervention) to a 2:1 individually matched historical prospective control group. HRQoL data were measured with the European Organization for Research and Treatment of Cancer Quality of Life Questionnaire-Core 30 at baseline (T0), 3 months (T1), and 6 months (T2) after start of treatment. Mixed effects linear regression models were used to compare HRQoL between the 2 groups over time.

**Results:**

From the 59 eligible patients for the CAPABLE intervention, 31 (53%) signed informed consent to participate. Baseline HRQoL was on average 10 points higher in the intervention group compared with controls, although equally matched on baseline and clinical characteristics. When correcting for sex, age, disease stage, and baseline scores, an adjusted difference in fatigue of −5.09 (95% CI −15.20 to 5.02, *P=*.32) at month 3 was found. No significant nor clinically relevant adjusted differences on other HRQoL domains over time were found. However, information satisfaction was significantly higher in the CAPABLE group (β=8.71, 95% CI 1.54‐15.88, *P*=.02).

**Conclusions:**

The intervention showed a limited effect on HRQoL, although there was a small improvement in fatigue at 3 months, as well as information satisfaction. When aiming at personalized patient and survivorship care, further optimization and prospective investigation of eHealth tools is warranted.

## Introduction

Immunotherapy with immune-checkpoint inhibitors (ICIs) and targeted therapies with BRAF/MEK inhibitors have significantly improved clinical outcomes for patients with melanoma and have become standard treatment for patients with high-risk and advanced disease [1-6]. Nevertheless, these novel systemic treatments are associated with short- and long-term (immune-related) adverse events (AEs) [7-10]. Furthermore, these AEs have shown to affect physical and psychosocial well-being of patients with melanoma [10-13]. Most prevalent in patients with melanoma undergoing immunotherapy with ICIs are cancer-related fatigue (CRF) and emotional distress, affecting both health-related quality of life (HRQoL) and clinical outcome to immunotherapy [8,14-16]. Efforts to address CRF include exercise recommendation, psychosocial support, mindfulness-based interventions, and yoga, showing positive effects on fatigue, emotional distress, and HRQoL [14,15,17-23].

Insufficient monitoring and reporting of AEs can exacerbate side effects, possibly leading to more frequent hospital visits and admissions [24-26]. Electronic symptom monitoring has shown to be associated with improved clinical outcomes such as survival and HRQoL in patients with cancer undergoing chemotherapy [27-30]. One way to improve patient care in immunotherapy could therefore involve regularly gathering patient-reported outcomes (PROs), such as symptom information, using patient-reported outcome measures (PROMs) through eHealth tools [31-34]. Furthermore, using biometric sensors could potentially detect symptoms and track physical activity in outpatient oncology settings [35,36]. To date, evidence of the effect of eHealth tools monitoring patients with melanoma on treatment with ICIs is scarce. One study showed that an electronic PROMs tool could not reduce the number of severe AEs, although it did increase HRQoL [34,37].

Health apps have also the potential to fulfill patients’ requirements for information and support, especially concerning symptom control and supportive services [38,39]. Furthermore, web-based programs and eHealth apps have incorporated nonpharmacological well-being interventions, such as promoting physical exercise, providing psychoeducation, mindfulness-based interventions, and yoga, to address CRF, showing encouraging outcomes [40,41]. By providing a combination of remote symptom monitoring, nonpharmacological well-being interventions, and information provision through an eHealth tool, patients believe this will positively affect their HRQoL and symptom burden [39].

Based on these insights, we previously developed a Cancer Patients Better Life Experience (CAPABLE) mobile app. CAPABLE is an extensively tested eHealth app as part of the EU Horizon 2020 program, designed to offer educational material, supportive care, remote symptom monitoring, and well-being interventions [42], initially for patients during and after ICIs, but open to treatment changes to targeted therapies. Development involved a user (patient)-centered design process in order to improve system usability and user acceptance [43]. The aim of this study was to explore the effect of CAPABLE on patient-reported outcomes, specifically fatigue and other HRQoL domains, compared with a matched historical control group receiving standard care [44].

## Methods

### Setting

The CAPABLE study was a prospective, exploratory, pilot study in which a cohort that received the CAPABLE smartphone app and a multisensory smartwatch (intervention) was compared with a historical prospective cohort that did not receive the CAPABLE app and smartwatch (control group). CAPABLE was registered as a medical device trial according to the Medical Device Regulation, article 62. A detailed description of the design of the pilot study and the design and development of the CAPABLE app was published previously [44]. Development and content was frozen during the trial. The CAPABLE app was used in its final operational state for the first time in a trial setting during this study, although preliminary prototype testing was done during system development. Briefly, participants included in the study were provided with the CAPABLE mobile app and a multisensorial smartwatch (ASUS VivoWatch 5 HC-B0) during the first 6 months of treatment with ICIs. The mobile app consisted of 3 main components; first, facilitating symptom and mental well-being monitoring, second, providing educational material, and finally, providing well-being interventions through goal setting and demonstrating the well-being intervention activity through a video or text and figures. The symptom monitoring functionality was used “as needed.” When a patient experienced a symptom, they were able to enter this into the CAPABLE app, upon which the decision support system managed the symptom [44]. No regular or static symptom monitoring was prompted by the app; however, the health care professional (HCP) monitored the symptom input coming from the patients on a daily basis and the information was included and discussed in clinical encounters. The well-being interventions could be executed from the home environment, and include a 30-minute walk, deep breathing practice, imagery training, physical activity of stretching, and strengthening exercises, Hatha Yoga or Nidra Yoga videos, or Tai Chi practice videos. The smartwatch collected data on heart rate, sleep (stages, hours, and performance), and physical activity, although data from the smartwatch were treated as ancillary data and not used for real-time symptom monitoring, decision support, or diagnosis. Over the course of the pilot study, participants were asked to complete PROMs at 3 time points. Results of the intervention (CAPABLE) group were compared with participants of 2 previously collected control groups (patient-reported outcomes in high risk and advanced melanoma patients cohort [PRO-MEL]; NL75996.031.20 and PROMs collected in clinical practice), which were 2 similar prospectively collected cohorts with the same inclusion criteria, but treated following standard of care and following the same follow-up schedule. PROMs in clinical practice were collected starting August 2024 and is still ongoing at the time of study. In addition, the PRO-MEL cohort was a prospective cohort that started inclusion in May 2021 and collected additional PROMs, as also collected in the CAPABLE cohort.

### Ethical Considerations

The Medical Ethical Committee NedMec (Amsterdam, the Netherlands) granted ethical approval (reference 22‐981/NL81970.000.22). The trial was prospectively registered at ClinicalTrials.gov (NCT05827289). Ethical approval also included the (secondary) use of data collected in the PRO-MEL and PROMs in clinical practice cohorts. Privacy and confidentiality protection was covered in the Medical Ethical Committee approval by a large data protection impact assessment. The study has not been amended during the course of the trial. Compensation to participants was not provided, except for the temporarily use of the smartphone and smartwatch used in the study.

### Recruitment

During a 6-month inclusion period, from April to October 2023, participants were recruited through their treating HCP and the CAPABLE research team recruited in an oncology-specialized center in Amsterdam, the Netherlands. The target sample for feasibility end points of this pilot was to include 36 patients, corresponding to 60 eligible patients and a 60% inclusion and compliance rate in the inclusion period [44]. Eligible participants had histologically confirmed stage III or IV melanoma and planned to start treatment with ICIs (anti–programmed-death 1 with or without anti–cytotoxic T-lymphocyte associated protein 4) according to standard clinical practice. Furthermore, participants had to be >18 years of age, had a sufficient understanding of the Dutch language, and were able to use a smartphone.

### Data Collection

Included patients were asked to use the CAPABLE app and smartwatch for a minimum period of 3 months and a maximum period of 6 months after start of treatment with ICIs. CAPABLE installation on mobile phones and baseline measurements took place after signing informed consent and before or during first ICI infusion. Research data were collected at baseline (T0), 3 months (T1), and 6 months (T2) after start of treatment by providing the participants a set of questionnaires. Clinical data (eg, staging, treatment details, and demographics) were extracted from the medical record during the study. PROMs data were stored and managed in ALEA (FormVision) [45]. Data generated through the CAPABLE app were stored on an internal secured drive.

To investigate the primary end point of this study, fatigue, the European Organization for Research and Treatment of Cancer (EORTC) Quality of Life Questionnaire-Core 30 (QLQ-C30) was used [46], a questionnaire developed to assess the quality of life of patients with cancer. Responses to this questionnaire range from 1 (not at all) to 4 (very much) and are linearly transformed into a functioning or symptom scale ranging from 0 to 100, with higher scores representing more experienced symptoms or a higher functioning, respectively. Primarily, the changes in fatigue over time in the intervention cohort were compared with the changes in fatigue over time in the control cohort. The validated fatigue scale of the QLQ-C30 is constructed out of 3 questions in the QLQ-C30 questionnaire, “Did you need to rest?”, “Have you felt weak?”, and “Were you tired?”. To explore secondary outcomes of this study, other domains of the EORTC QLQ-C30 were investigated (functioning and other symptom scales), as well as changes between the CAPABLE and (matched) control group when looking at the EuroQol 5D (EQ-5D-5L) [47], Functional Assessment of Cancer Therapy-Melanoma (FACT-M) [48], and EORTC Quality of Life Questionnaire-Information 25 (QLQ-INFO25) [49].

Feasibility outcomes were investigated throughout the course of this pilot study by exploring the inclusion and compliance rate of the CAPABLE app users. Recruitment rate was calculated as the percentage of patients included in the study out of the patients screened for eligibility. Patient compliance was calculated as the percentage of patients completing the questionnaires per follow-up moment. Finally, patient retention was calculated as the percentage of patients adhering to the CAPABLE mobile app for 6 months (ie, ≥1 interaction with any of the functionalities within the follow-up period). Patient engagement with the app was presented by descriptive data on the use of the symptom reporting and well-being intervention functionalities. Extensive data collection methods and corresponding figures and tables are described in the previously published study protocol [44].

### Data Analysis

Patients that completed at least 1 PROM over the course of the study were included in the final analysis. Because of low inclusion in the (control cohort) PRO-MEL study, matching was done on a control group composed of patients from both the PRO-MEL and PROMs in clinical practice cohort who filled in the EORTC QLQ-C30, FACT-M, and EQ-5D-5L according to the same follow-up schedule. Patients in the CAPABLE cohort were individually matched 1:2 with patients in the control cohorts based on sex, age, and tumor staging. To compare information needs (QLQ-INFO25) between the CAPABLE cohort and controls, no matching was performed, and comparison consisted of the entire PRO-MEL cohort to increase statistical power and be able to interpret results.

Descriptive statistics were calculated to provide information about the patient population, feasibility, and engagement with the CAPABLE app. For the purpose of this study, mean scores for fatigue and other QLQ-C30 domains were calculated and presented according to current guidelines [50]. To compare the mean fatigue scores and other HRQoL outcomes between the group receiving the CAPABLE intervention and the control group at each individual time point, independent sample *t* tests were used. To analyze the differences in all outcomes on different time points between the CAPABLE cohort and matched controls over time, linear mixed effects models were used. Statistical models were adjusted for age, sex, tumor stage, time, and baseline scores (with an interaction term between time and cohort). A 2-tailed *P* value<.05 was considered statistically significant, although *P* values in this pilot setting were not powered to provide much information due to the small sample size. Therefore, this study mostly focused on clinically relevant differences according to Cocks et al [51]. Similar methods were used for analyzing the EQ-5D-5L, FACT-M, and QLQ-INFO25.

Missing items from the questionnaires were imputed according to corresponding EORTC guidelines [50]. The scale scores of the EORTC QLQ-C30 were set to missing if fewer than half of the items on a given scale were answered. Where at least 50% of the relevant scale scores were present, the missing values were replaced by the mean of the present values. We applied the same strategy to the other questionnaires as no other guidelines are available for those. Statistical analyses and matching procedures were done using Stata version 15 (StataCorp) [52].

## Results

### Overview

**Figure 1.**
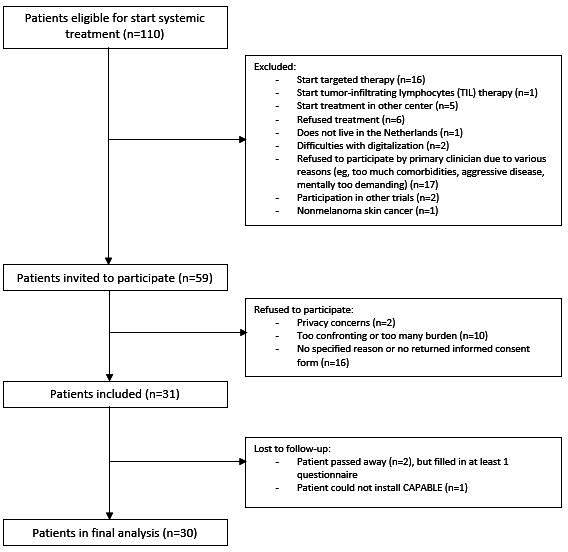
Cancer Patients Better Life Experience (CAPABLE) study inclusion flowchart.

In total, 110 patients were screened for eligibility for the CAPABLE trial in the 6-month inclusion period (Figure 1). Main reasons for noneligibility were the start of targeted therapy instead of ICIs (n=16) or the patient not being invited for inclusion by the treating physician’s decision (eg, aggressive disease, comorbidities, symptomatic brain metastasis, low health literacy, and mentally too demanding; n=17). Eventually, 59 out of 110 (54%) patients were contacted to participate. Most of the contacted patients who did not return a consent form did not specify a reason (n=16). Reasons specified for not participating in the pilot study included privacy concerns (n=2) and the expectation that the burden would be too high (n=10). Of the 31 included patients, 1 did not manage to install the CAPABLE app before T1. A total of 30 patients were taken into consideration for the statistical analysis, although 2 patients died due to progressive disease before reaching T2. In total, 297 patients (70 from the PRO-MEL cohort and 227 from the PROMs in clinical practice cohort) were eligible for individual matching. This yielded 56 patients that were matched 2:1 to the CAPABLE cohort. Thus, a group of 86 patients was included in the analysis of the EORTC QLQ-C30, FACT-M, and EQ-5D-5L. Since data on the EORTC QLQ-INFO25 were only available in the PRO-MEL cohort, the entire PRO-MEL cohort (n=70) was used in the comparison with the CAPABLE group (n=30) for these secondary end points.

Both cohorts were equally balanced in terms of age, sex, and tumor stage, due to matching (Table 1). Median age of the CAPABLE cohort was 65 (IQR 55‐72) years. Females represented 61% (19/31) of the included participants, and approximately half the participants had stage IV disease (17/31, 55%). Most patients received anti-PD1 monotherapy in the CAPABLE and matched control group (19/31, 61% and 37/56, 66%, respectively). In the matched control group, 21% (12/56) of patients received targeted therapy after therapy switch, compared with 7% (2/31) in the CAPABLE group, but this difference was not statistically significant (*P=*.07).

**Table 1.** Participants’ clinical characteristics. Not all percentages add up to 100% as multiple patients received multiple treatments. Missing data were not taken into consideration when calculating the *P* values.

Characteristic		CAPABLE^a^ cohort (n=31)	Control cohort (n=56)	*P* value^b^
**Sex, n (%)**				.96
	Male	12 (39)	22 (39)	
	Female	19 (61)	34 (61)	
Age (years), median (IQR)		65 (55-72)	64 (56-71)	.99
**Tumor stage, n (%)**				.96
	III	14 (45)	25 (45)	
	IV	17 (55)	31 (55)	
**Treatment, n (%)**				
	Anti-PD-1^c^	19 (61)	37 (66)	.66
	Anti-CTLA-4^d^ + Anti-PD-1	14 (45)	23 (41)	.71
	Radiotherapy	6 (19)	12 (21)	.82
	Surgery before ICIs^e^	10 (32)	23 (41)	.42
	Targeted therapy	2 (7)	12 (21)	.07
**Treatment line, n (%)**				.90
	1	24 (77)	44 (79)	
	>1	7 (23)	12 (21)	

a CAPABLE: Cancer Patients Better Life Experience

b *P* values are based on *χ*2 tests for group variables and Mann-Whitney test for age.

c PD-1: programmed-death 1.

d CTLA-4: cytotoxic T-lymphocyte associated protein 4.

e ICI: immune-checkpoint inhibitor.

### Fatigue

The trend in unadjusted fatigue over time was similar between the CAPABLE group and the matched controls although fatigue seems to increase less in the CAPABLE cohort (Figure S1 in Multimedia Appendix 1). A 5-point difference was shown in baseline fatigue. The CAPABLE group had lower fatigue score on baseline compared with the matched controls (mean 18.4, SD 21.7) compared with the matched controls (mean 23.4, SD 19.4; *P*=.28), increasing to a difference of 8.2 points at month 3 (mean 23.0, SD 25.2 vs mean 31.2, SD 24.1; *P*=.17) (Table S1 in Multimedia Appendix 1). When correcting for sex, age, stage, time, and baseline scores, an adjusted difference in fatigue of −5.09 (95% CI −15.20 to 5.02; *P=*.32) for the CAPABLE group at month 3 was observed (Table 2). Although this result was not statistically significant, this difference was considered a small, clinically relevant difference. At month 6, a nonsignificant and nonclinically relevant difference was shown between the 2 groups (β=−2.32, 95% CI −12.81 to 8.16; *P*=.66).

**Table 2.** Adjusted mixed effects linear regression analysis on fatigue as measured by the Quality of Life Questionnaire-Core 30 (QLQ-C30) between Cancer Patients Better Life Experience (CAPABLE) group and matched controls over time.

Fatigue		β (95% CI)	*P* value	Clinical relevance
**Cohort**				
	Controls	Ref	—^a^	—
	CAPABLE	−1.43 (−9.00 to 6.13)	.71	Trivial
**Sex**				
	Male	Ref	—	—
	Female	1.31 (−4.06 to 6.68)	.63	Trivial
Age		0.03 (−0.22 to 0.27)	.83	Trivial
**Stage**				
	Stage III	Ref	—	—
	Stage IV	−0.35 (−5.36 to 4.66)	.89	Trivial
**Time**				
	Baseline	Ref	—	—
	Month 3	8.28 (2.38 to 14.18)	.01	Small
	Month 6	5.60 (−0.34 to 11.54)	.07	Small
**Cohort×time**				
	Controls × baseline	Ref	—	—
	CAPABLE × month 3	−5.09 (−15.20 to 5.02)	.32	Small
	CAPABLE × month 6	−2.32 (−12.81 to 8.16)	.66	Trivial
Baseline score		0.72 (0.58 to 0.85)	<.001	— (offset)

a Not applicable.

### Health-Related and Melanoma-Specific Quality of Life

A significant difference in baseline scores was observed between the 2 cohorts for most of the HRQoL domains measured by the EORTC QLQ-C30 (Table S1 in Multimedia Appendix 1). On all functioning scales except cognitive functioning, the CAPABLE group reported better function in terms of both statistical significance and clinical relevance, with mean baseline differences ranging from 8.5 in social functioning (*P=*.11) to 12.0 in role functioning (*P=*.07) (Table S1 in Multimedia Appendix 1). After adjusting for covariates in multivariable regression analysis, no statistically significant nor clinically relevant changes on any of the HRQoL domains were observed between the CAPABLE group and matched controls at either month 3 or month 6 follow-up (Table 3).

**Table 3.** Adjusted mixed effects linear regression analyses on different health-related quality of life (HRQoL) outcomes as measured by the European Organization for Research and Treatment of Cancer (EORTC) Quality of Life Questionnaire-Core 30 (QLQ-C30) between Cancer Patients Better Life Experience (CAPABLE) group and matched controls over time. Analyses are adjusted for age, sex, stage, and baseline scores.

HRQoL subscales		β (95% CI)^a^	*P* value	Clinical relevance
**Physical functioning**				
	CAPABLE × month 3	−4.45 (−11.93 to 3.03)	.24	Trivial
	CAPABLE × month 6	−6.31 (−14.09 to 1.47)	.11	Small
**Role functioning**				
	CAPABLE × month 3	0.55 (−11.88 to 12.99)	.93	Trivial
	CAPABLE × month 6	−3.82 (−16.74 to 9.10)	.56	Trivial
**Emotional functioning**				
	CAPABLE × month 3	−5.77 (−14.71 to 3.16)	.21	—^b^
	CAPABLE × month 6	−8.41 (−17.63 to 0.80)	.07	—
**Social functioning**				
	CAPABLE × month 3	−1.89 (−12.54 to 8.77)	.73	Trivial
	CAPABLE × month 6	1.09 (−9.95 to 12.13)	.85	Trivial
**Cognitive functioning**				
	CAPABLE × month 3	0.30 (−7.16 to 7.77)	.94	Trivial
	CAPABLE × month 6	1.06 (−6.64 to 8.77)	.79	Trivial
**Insomnia**				
	CAPABLE × month 3	3.65 (−1.68 to 13.93)	.58	Trivial
	CAPABLE × month 6	0.88 (−12.57 to 14.33)	.90	Trivial
**Financial difficulties**				
	CAPABLE × month 3	4.93 (−0.33 to 10.20)	.07	Small
	CAPABLE × month 6	7.47 (1.99 to 12.95)	.01	Small
**Summary score**				
	CAPABLE × month 3	−3.37 (−8.78 to 2.04)	.22	—
	CAPABLE × month 6	1.19 (−4.40 to 6.80)	.68	—

a Results presented are adjusted βs coming from interaction between cohort and time. Matched controls at baseline are reference group.

b Not applicable.

Similar baseline differences were observed in melanoma-specific quality of life as measured by the melanoma subscale (MS) and melanoma surgery subscale (MSS) of the FACT-M. Melanoma-specific quality of life showed a significant baseline difference between the CAPABLE group and matched controls when looking at both the MS (mean 57.5, SD 5.3 vs mean 50.4, SD 8.3; *P*<.001) and the MSS (mean 28.1, SD 4.4 vs mean 22.4, SD 6.3; *P*<.001). Mean scores did not change much over time in both cohorts (Table S3 in Multimedia Appendix 1). When adjusted for sex, age, tumor stage, time, and baseline scores, there were also no significant changes in melanoma-specific quality of life between both cohorts over time (Table 4). Utility scores of the EQ-5D-5L yielded similar results in terms of mean scores, and when adjusted for all covariates, no effect of CAPABLE was seen compared with matched controls (Table S3 in Multimedia Appendix 1; Table 4). However, HRQoL as measured by the visual analog scale, was significantly higher for the CAPABLE cohort compared with the matched controls on month 3 and month 6 when corrected for sex, age, and stage of disease (B=10.28, 95% CI 1.45‐19.11, *P*=.02 and B=11.50, 95% CI 2.08‐20.92, *P=*.017, respectively).

**Table 4.** Adjusted univariable mixed effects linear regression analyses on different patient reported outcome measures between CAPABLE group and matched controls. Analyses are adjusted for age, sex, stage, and baseline scores.

HRQoL^a^ subscales			β (95% CI)^b^	*P* value
**FACT-M^c^**				
	**MS^d^ (range 0-64)**			
		CAPABLE^e^×month 3	−1.54 (−5.37 to 2.29)	.43
		CAPABLE×month 6	−0.82 (−4.78 to 3.14)	.69
	**MSS^f^ (range 0-32)**			
		CAPABLE×month 3	0.91 (−2.34 to 4.17)	.58
		CAPABLE×month 6	−0.68 (−4.00 to 2.64)	.69
**EQ-5D-5L**				
	**Utility (range 0-1)**			
		CAPABLE×month 3	−0.05 (−0.11 to 0.01)	.10
		CAPABLE×month 6	−0.03 (−0.10 to 0.03)	.33
	**VAS^g^ (range 0-100)**			
		CAPABLE×month 3	10.28 (1.45 to 19.11)	.02
		CAPABLE×month 6	11.50 (2.08 to 20.92)	.02

a HRQoL: health-related quality of life.

b Results presented are adjusted βs coming from interaction between cohort and time. Matched controls at baseline are reference group.

c FACT-M: Functional Assessment of Cancer Therapy-Melanoma.

d MS: melanoma subscale.

e CAPABLE: Cancer Patients Better Life Experience.

f MSS: melanoma surgery subscale.

g VAS: visual analog scale.

### Information Needs

Overall, information provision as reported by the EORTC QLQ-INFO25 was significantly lower in the control cohort on both baseline and month 6 (Table S4 in Multimedia Appendix 1). When adjusting for age, sex, and baseline scores, no separate domains showed significant improvements of the CAPABLE cohort. However, information satisfaction was significantly higher in the CAPABLE cohort (B=8.71, 95% CI 1.54‐15.88; *P*=.02) (Table 5).

**Table 5.** Adjusted univariable mixed effects linear regression analyses on information domains between CAPABLE group and PRO-MEL controls. Analyses are adjusted for age, sex, stage, and baseline scores.

Information domains^a^	β (95% CI)	*P* value
Disease	3.38 (−5.49 to 12.25)	.46
Medical tests	9.35 (0.22 to 18.48)	.05
Treatment	1.59 (−6.89 to 10.06)	.71
Other services	2.46 (−7.28 to 12.19)	.62
Different places of care	−2.19 (−14.04 to 9.67)	.72
Things you can do to help yourself	−6.12 (−15.54 to 3.30)	.20
Satisfaction with information received	8.71 (1.54 to 15.88)	.02
Overall the information has been helpful	3.69 (−4.77 to 12.14)	.39

a Controls are reference group.

### Feasibility

Because of project time constraints, only 31 of the planned 36 patients were included in this study. In total, 59 patients were eligible for study participation, resulting in a recruitment rate of 53% in the set period, whereas the planned recruitment rate for reaching the feasibility end point was 60%. However, patient compliance and patient retention remained high in the patients that were included. Patient compliance to the PROMs at baseline was 98% (30/31), at T1 was 90% (27/30), and at T2 was 79% (22/28). Finally, 27 out of 31 (87%) patients adhered to using the CAPABLE app until at least T1, which dropped to 24 patients (77%) using the app at T2. Furthermore, 2 of those patients died because of rapidly progressive disease during the trial. Adherence to smartwatch use was lower with only 43% (13/30) usage at T2 due to smartwatch issues. In total, 27 individual problems with the CAPABLE app and smartwatch were reported during the trial, mostly in the first 3 months of usage. Almost half of patients (14/30, 47%) reported at least 1 problem with the CAPABLE app or one of its functionalities. The majority of reported problems were related to login issues (7/20, 35%), smartwatch problems (communication between app and discomfort of the smartwatch; 8/20, 41%), and problems with the symptom reporting workflow (3/20, 15%).

### Engagement With the System

Concerning the symptom reporting functionality, 18 out of 30 patients have actively used the CAPABLE app and reported at least 1 distinct symptom or symptom episode (range 1‐7) (Figure S2 in Multimedia Appendix 1). In total, 20 distinct immune-related AEs were reported through the CAPABLE app, with reports of 33 grade 1, 28 grade 2, 17 grade 3, and 3 grade 4 symptoms according to the mapped Common Terminology Criteria for AEs version 5 (Table S5 in Multimedia Appendix 1). Symptom episodes ranged from 1.5 days for headache to 149.6 days for muscle pain (Table S6 in Multimedia Appendix 1).

Engagement with the well-being interventions was on average lower than symptoms reporting (Table S7 in Multimedia Appendix 1). Interventions were not prescribed by HCPs and were free to use by the patients. The intervention related to taking a walk was the most often used, with 327 execution times, reported by 9 distinct patients. Some patients used the other interventions infrequently. Furthermore, 8 out of 30 patients used the interventions more than 5 times (range 8‐213). Interventions that were used infrequently were mainly used during the first weeks of enrollment, suggesting that the interventions were tried out at the start of the pilot. The walking intervention was executed throughout the course of the pilot study, with more engagement in summer than in fall and winter (October-December).

## Discussion

### Principal Findings

The aim of this pilot study was to explore the effect of the CAPABLE mobile app on patient-reported outcomes, specifically fatigue, in patients with melanoma starting ICIs, compared with a historical control group receiving standard care. Our results showed no significant adjusted differences on fatigue and other HRQoL domains between the CAPABLE cohort and matched controls during the first 6 months of treatment. However, although not statistically significant, we did find that the CAPABLE cohort reported a smaller relevant increase in fatigue at month 3 follow-up compared with matched controls. Furthermore, information satisfaction was significantly higher in the CAPABLE users. The secondary goal of this study was to show patients’ acceptance and feasibility of the CAPABLE app. With an observed recruitment rate of 53%, our feasibility end point of 60% was not met. A third of the patients refused to participate because study and questionnaires were expected to be too burdensome. Furthermore, HCPs did not feel comfortable including patients in the study because they expected it to be too burdensome for some patients (based on oral feedback). Furthermore, technology barriers might have played a role, as observed in other studies with eHealth apps [53].

We observed a significant baseline difference in almost all the HRQoL domains between the CAPABLE cohort and matched controls, suggesting a selection bias. Although matched on baseline characteristics, HRQoL domains in the CAPABLE group are clinically relevantly and significantly higher than in the matched controls. Therefore, results obtained in the CAPABLE group could have been influenced by the phenomenon “regression towards the mean,” as improvement of HRQoL was almost impossible to achieve [54]. However, this phenomenon looks like it occurred in all HRQoL domains, except for fatigue, as we see an improvement only in fatigue for the CAPABLE cohort, although not statistically significant. This observation also underscores the importance of including patients with lower HRQoL in interventions designed to improve this outcome, for example, by minimizing the expected burden of participation [55].

Several studies have shown the benefits of eHealth on CRF. Supporting the small (nonsignificant) difference found in fatigue in our study, a meta-analysis done on 9 studies showed a statistically significant beneficial effect of eHealth interventions on CRF [23]. Furthermore, these eHealth tools were mostly designed to target CRF solely and did not have the multimodal aspects of our CAPABLE app. Furthermore, we were not able to disentangle which specific functionality was responsible for possible changes in fatigue, or if it was a combination of all functionalities. In addition, our sample size was not large enough to provide statistical significance; our pilot study was designed to provide descriptive statistics and focused largely on clinical relevance [51].

A similar study in Denmark, with electronic symptom monitoring carried out in patients with metastatic melanoma starting treatment with ICIs yielded improved HRQoL in the intervention group, as measured by the FACT-M and EQ-5D-5L [34,37], although the differences Tolstrup et al [37] obtained were also not clinically relevant. The Danish study was a randomized controlled trial (RCT) and had an active weekly symptom-monitoring component by their HCP; components of the trial that could have influenced the results compared with passive HCP monitoring in our real-world single-arm setting [56,57].

Information satisfaction was significantly higher in the CAPABLE cohort compared with the control group. Studies done on information provision through eHealth tools in the Dutch cancer care are still conflicting [58]. For example, in an RCT investigating a web-based eHealth app to support multiple cancer patient groups, improvement of knowledge was not reached [59]. However, another study showed that higher information satisfaction might contribute to patient knowledge and decision involvement [60]. Therefore, an eHealth tool, such as CAPABLE, might still support patient knowledge and shared decision-making.

Results of our study might have been influenced by barriers when integrating and implementing eHealth into clinical practic. Although the CAPABLE system was developed using all relevant stakeholders, the CAPABLE app was not integrated in our electronic health record, causing our HCPs to work with 2 different systems, leading to resistance. In-depth results of user experience and usability research done in HCPs still need to be analyzed, but verbal feedback suggested this was a large barrier for monitoring patients with this app. To date, successful implementation and use of digital health interventions remains limited worldwide by integration into electronic health records, impairing the possible positive effect of such interventions [53]. Second, an existing challenge in digital health interventions research is the recruitment of target populations in need of such interventions [53,61,62]. Both not reaching our feasibility end point of 60% and high HRQoL baseline scores (probably because of selection bias) confirm this challenge. Consequently, results of this study need to be interpreted with caution and future research should make more effort into recruitment strategies including weaker populations, as well as considering health literacy. Furthermore, efforts are needed to reduce patient burden in this type of studies, both in terms of intervention as with research questionnaires, as it is essential to include all patients with serious health conditions [55].

### Limitations

Several limitations of this study need to be considered when interpreting the results. Our sample size was too small to prove significant differences on HRQoL outcomes. Because of time constraints of our project, whose main focus was the design and development of the system, inclusion period was short and only 31 patients out of the anticipated 36 were included. Second, most of the control group was collected during the COVID-19 pandemic, which might have influenced HRQoL outcomes in these patients. However, a study done by van de Poll-Franse et al [63] showed that the crisis might have affected well-being of general population more than in that of cancer patients. In addition, another negative aspect of the COVID-19 pandemic was related to some delays in the software development for the CAPABLE eHealth app in an already-restricted project timeframe, which might have resulted in an increase in app issues reported by patients. While in-depth usability outcomes of this study have yet to be analyzed, we observed a relatively high proportion of technical problems during our study and we gathered multiple areas of improvement from verbal feedback from patients, which could have affected the results. Another limitation of the study was our decision to use a matched historical control cohort rather than an RCT. An RCT would have allowed better isolation of the effect of using the app. However, use of the historical cohort allowed us to recognize and characterize the participation bias in the intervention participants. This bias may have contributed to the lack of a clinically relevant effect in our main outcome.

However, a large strength of this study has been that CAPABLE was developed with co-design of patients and HCPs, following user-centered design principles, starting with explorative interviews and undergoing 3 testing rounds. Furthermore, our study was conducted in the real-world setting of the pilot trial; included patients were treated according to clinical practice. The CAPABLE app was added as a monitoring and coaching system tool and could intervene in management of patients after severe symptom reports. Due to the real-world setting of this trial, results on PROs, feasibility, and engagement with eHealth might be more generalizable to real-world patients compared with results found in clinical trials.

Future and larger studies could benefit from including patients more inclusively in terms of low health literacy and social economic status. Inclusion criteria should be broadened to reduce ceiling effects at baseline. Furthermore, the setting of this pilot study might have played a pivotal role, as we included patients in a dedicated cancer and melanoma center with a lot of ongoing clinical trials and other intervention studies and also standard care including easily accessible specialized nurse practitioners.

### Conclusion

In our small, nonrandomized study we were unable to show that mobile-based coaching and follow-up affected HRQoL significantly, although results suggest a small clinical improvement of fatigue at 3 months follow-up in the app users. Ceiling effects due to large baseline differences might have caused the impact of CAPABLE to be negligible for patients with higher baseline HRQoL. CAPABLE resulted in significantly higher information satisfaction compared with controls. Although the feasibility end point of 60% was not met, adherence to the system was high. Further optimization of CAPABLE, taking into account patient-related and technology-related barriers is needed before future investigation in an RCT and might influence HRQoL end points. Furthermore, when aiming at personalized patient and survivorship care, further optimization and prospective investigation of eHealth tools is warranted.

## Supplementary material

10.2196/58938 Multimedia Appendix 1 Supplementary tables and figures.

## Abbreviations

AE: adverse event
CAPABLE: Cancer Patients Better Life Experience
CRF: cancer-related fatigue
EORTC: European Organization for Research and Treatment of Cancer
EQ-5D-5L: EuroQol-5D-5L
FACT-M: Functional Assessment of Cancer Therapy-Melanoma
HCP: health care professional
HRQoL: health-related quality of life
ICI: immune-checkpoint inhibitor
MS: melanoma subscale
MSS: melanoma surgery subscale
PRO: patient-reported outcome
PRO-MEL: patient-reported outcomes in high risk and advanced melanoma patients cohort
PROM: patient-reported outcome measure
QLQ-C30: Quality of Life Questionnaire-core 30
QLQ-INFO25: Quality of Life Questionnaire-Information 25
RCT: randomized controlled trial
