# Supplementary material for: Exploring the Impact of the Multimodal CAPABLE eHealth Intervention on Health-Related Quality of Life in Patients With Melanoma Undergoing Immune-Checkpoint Inhibition: Prospective Pilot Study
Source: JMIR Cancer. 2025 Jan 30;11:e58938. doi: 10.2196/58938 (PMC11800704; doi:10.2196/58938)
Supplement: Multimedia Appendix 1 [file cancer-v11-e58938-s001.docx]

**Supplementary material – Exploring the impact of the multimodal CAPABLE eHealth intervention on health-related quality of life in melanoma patients undergoing immune-checkpoint inhibition: A prospective pilot study**


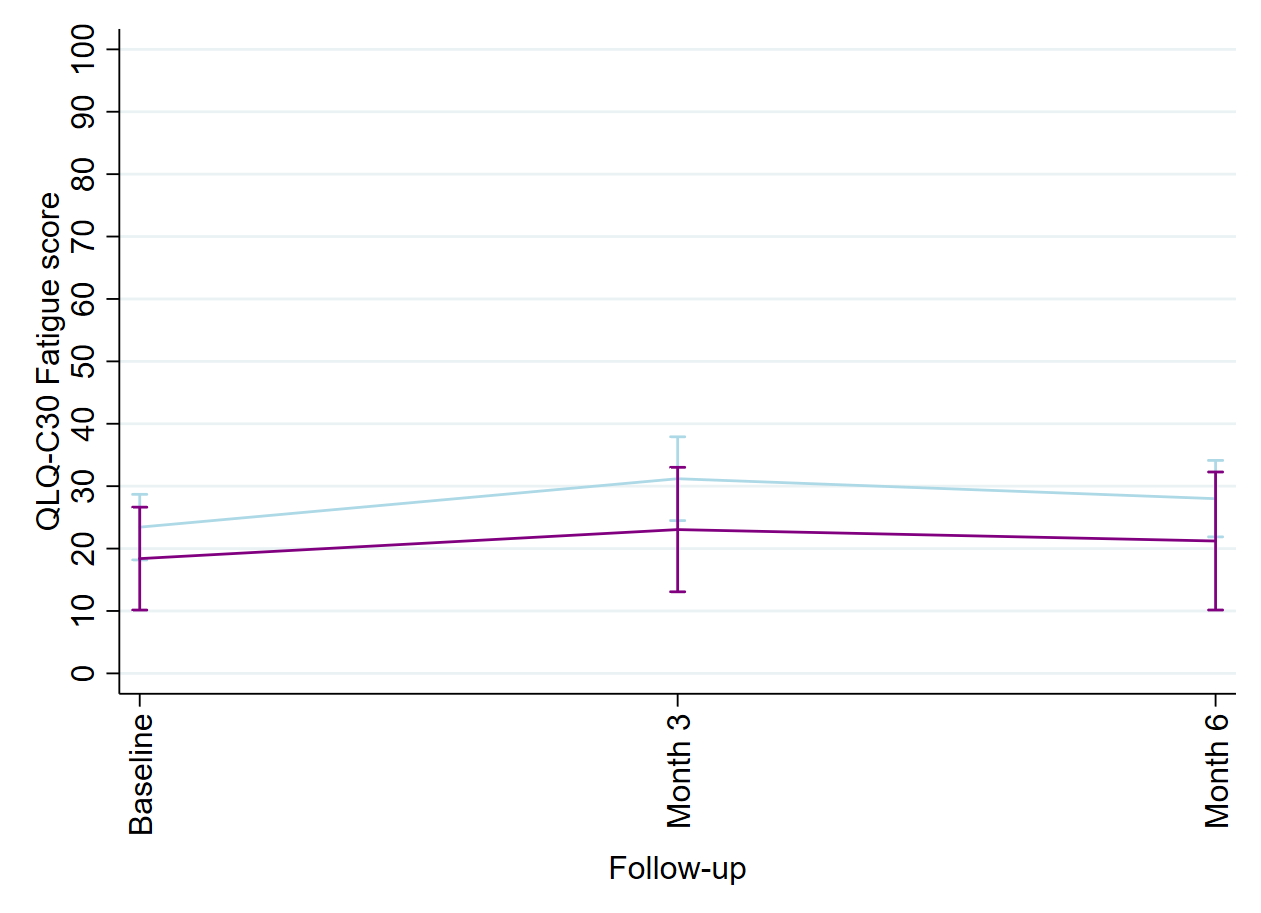


**Figure S1. Unadjusted mean fatigue score over time by subgroups, as measured by the EORTC QLQ-C30. Light blue line represents the patients in the matched control cohort. Purple line represents the patients in the CAPABLE cohort.**

| **Table S1. Unadjusted mean HRQoL scores per subgroup over time as measured by the EORTC QLQ-C30** | | | | |
| --- | --- | --- | --- | --- |
| **Subscale, mean ± SD** | **CAPABLE cohort^1^** | **Matched controls^2^** | **Difference** | **p-value^a^** |
| Fatigue |  |  |  |  |
| Baseline | 18.4 ± 21.7 | 23.4 ± 19.4 | -5.0 | 0.281 |
| Month 3 | 23.0 ± 25.2 | 31.2 ± 24.1 | -8.2 | 0.165 |
| Month 6 | 21.2 ± 24.9 | 28.0 ± 21.6 | -6.8 | 0.245 |
| Physical functioning |  |  |  |  |
| Baseline | 91.3 ± 12.6 | 81.9 ± 21.4 | 9.3 | 0.034 |
| Month 3 | 89.4 ± 17.1 | 85.4 ± 16.7 | 4.0 | 0.320 |
| Month 6 | 90.9 ± 16.8 | 86.8 ± 15.7 | 4.1 | 0.318 |
| Role functioning |  |  |  |  |
| Baseline | 86.2 ± 26.8 | 74.2 ± 29.4 | 12.0 | 0.071 |
| Month 3 | 87.0 ± 19.8 | 75.3 ± 25.2 | 11.7 | 0.039 |
| Month 6 | 87.1 ± 23.5 | 78.3 ± 25.7 | 8.8 | 0.175 |
| Emotional functioning |  |  |  |  |
| Baseline | 81.3 ± 17.3 | 70.4 ± 16.6 | 10.9 | 0.001 |
| Month 3 | 82.1 ± 25.1 | 78.7 ± 20.4 | 3.4 | 0.527 |
| Month 6 | 83.3 ± 18.0 | 81.5 ± 17.8 | 1.8 | 0.689 |
| Social functioning |  |  |  |  |
| Baseline | 87.9 ± 21.8 | 79.4 ± 24.0 | 8.5 | 0.114 |
| Month 3 | 87.7 ± 23.4 | 82.1 ± 23.3 | 5.6 | 0.315 |
| Month 6 | 93.2 ± 11.2 | 83.7 ± 21.2 | 9.5 | 0.053 |
| Cognitive functioning |  |  |  |  |
| Baseline | 90.8 ± 17.0 | 89.4 ± 15.8 | 1.4 | 0.706 |
| Month 3 | 84.0 ± 22.8 | 83.7 ± 21.3 | 0.3 | 0.951 |
| Month 6 | 88.6 ± 14.0 | 85.0 ± 17.3 | 3.6 | 0.387 |
| Insomnia |  |  |  |  |
| Baseline | 11.5 ± 18.4 | 26.5 ± 23.7 | -15.0 | 0.004 |
| Month 3 | 14.1 ± 23.4 | 25.0 ± 27.1 | -10.9 | 0.085 |
| Month 6 | 10.6 ± 17.4 | 25.3 ± 27.4 | -14.7 | 0.029 |
| Financial score |  |  |  |  |
| Baseline | 4.6 ± 14.7 | 6.1 ± 13.0 | -1.5 | 0.640 |
| Month 3 | 6.2 ± 13.2 | 3.2 ± 9.9 | 3.0 | 0.265 |
| Month 6 | 9.1 ± 15.2 | 2.6 ± 9.1 | 6.5 | 0.027 |
| Summary score |  |  |  |  |
| Baseline | 90.6 ± 10.3 | 82.9 ± 11.8 | 7.7 | 0.005 |
| Month 3 | 86.0 ± 17.3 | 83.5 ± 13.2 | 2.4 | 0.499 |
| Month 6 | 92.6 ± 9.9 | 84.0 ± 11.8 | 8.6 | 0.005 |
| ^a^Two-sample t-tests were used to determine p-values corresponding to the effect size  ^1^CAPABLE group: baseline N=29, month 3 N=27 , month 6 N=22  ^2^ Control group: baseline N=55, month 3 N=52, month 6 N=51 | | | | |

**Table S3. Mean (melanoma specific) HRQoL and utility scores over time for both CAPABLE group and matched controls as measured by the FACT-M and EQ-5D-5L**

| **Domain mean ± SD** | **CAPABLE cohort^1^** | **Matched controls^2^** | **p-value** |
| --- | --- | --- | --- |
| *FACT-M* |  |  |  |
| MS (range: 0-64) |  |  |  |
| Baseline | 57.5 ± 5.3 | 50.4 ± 8.3 | <0.001 |
| Month 3 | 56.3 ± 7.6 | 51.2 ± 7.8 | 0.006 |
| Month 6 | 57.7 ± 5.8 | 50.8 ± 8.4 | <0.001 |
| MSS (range: 0-32) |  |  |  |
| Baseline | 28.1 ± 4.4 | 22.4 ± 6.3 | <0.001 |
| Month 3 | 28.8 ± 2.9 | 22.9 ± 6.1 | <0.001 |
| Month 6 | 28.5 ± 3.6 | 22.6 ± 6.7 | <0.001 |
| *EQ-5D-5L* |  |  |  |
| Utility (range: 0-1) |  |  |  |
| Baseline | 0.88 ± 0.13 | 0.79 ± 0.15 | 0.008 |
| Month 3 | 0.86 ± 0.13 | 0.82 ± 0.15 | 0.251 |
| Month 6 | 0.91 ± 0.12 | 0.84 ± 0.14 | 0.056 |
| VAS (range: 0-100) |  |  |  |
| Baseline | 75.7 ± 21.9 | 76.3 ± 14.6 | 0.878 |
| Month 3 | 79.0 ± 19.0 | 71.4 ± 18.5 | 0.087 |
| Month 6 | 85.1 ± 16.8 | 74.5 ± 12.6 | 0.006 |
| ^1^CAPABLE group: baseline N=29, month 3 N=27 , month 6 N=22  ^2^ Control group: baseline N=58, month 3 N=55, month 6 N=54  MS: melanoma subscale, MSS: melanoma surgery subscale, VAS: visual analog scale | | | |

**Table S4. Mean information satisfaction scores over time for both CAPABLE group and complete unmatched PRO-MEL group as measured by the EORTC INFO25**

| **Information domain, mean ± SD** | **CAPABLE cohort^1^** | **PRO-MEL cohort^2^** | **p-value** |
| --- | --- | --- | --- |
| Disease |  |  |  |
| Baseline | 69.1 ± 18.9 | 61.5 ± 22.0 | 0.120 |
| Month 6 | 72.2 ± 23.9 | 58.9 ± 23.8 | 0.059 |
| Medical tests |  |  |  |
| Baseline | 79.0 ± 20.3 | 73.5 ± 21.7 | 0.261 |
| Month 6 | 78.3 ± 22.9 | 65.1 ± 24.5 | 0.061 |
| Treatment |  |  |  |
| Baseline | 66.7 ± 21.6 | 58.9 ± 16.6 | 0.068 |
| Month 6 | 62.2 ± 22.5 | 51.6 ± 19.4 | 0.084 |
| Other services |  |  |  |
| Baseline | 38.3 ± 22.4 | 28.3 ± 21.7 | 0.051 |
| Month 6 | 40.4 ± 28.9 | 25.9 ± 19.0 | 0.041 |
| Different places of care |  |  |  |
| Baseline | 32.2 ± 28.8 | 19.9 ± 25.9 | 0.045 |
| Month 6 | 26.7 ± 33.5 | 17.3 ± 23.3 | 0.263 |
| Things you can do to help yourself |  |  |  |
| Baseline | 44.0 ± 30.2 | 28.0 ± 27.1 | 0.014 |
| Month 6 | 45.0 ± 27.1 | 34.5 ± 23.1 | 0.156 |
| Satisfaction with the information received |  |  |  |
| Baseline | 80.5 ± 16.7 | 73.8 ± 23.9 | 0.181 |
| Month 6 | 80.0 ± 16.8 | 65.5 ± 21.2 | 0.014 |
| Overall the information has been helpful |  |  |  |
| Baseline | 86.2 ± 16.7 | 79.4 ± 19.3 | 0.104 |
| Month 6 | 85.7 ± 19.9 | 72.6 ± 18.2 | 0.021 |
| ^1^CAPABLE group: baseline N=28, month 6 N=20  ^2^ PRO-MEL group: baseline N=63, month 6 N=28 | | | |

**
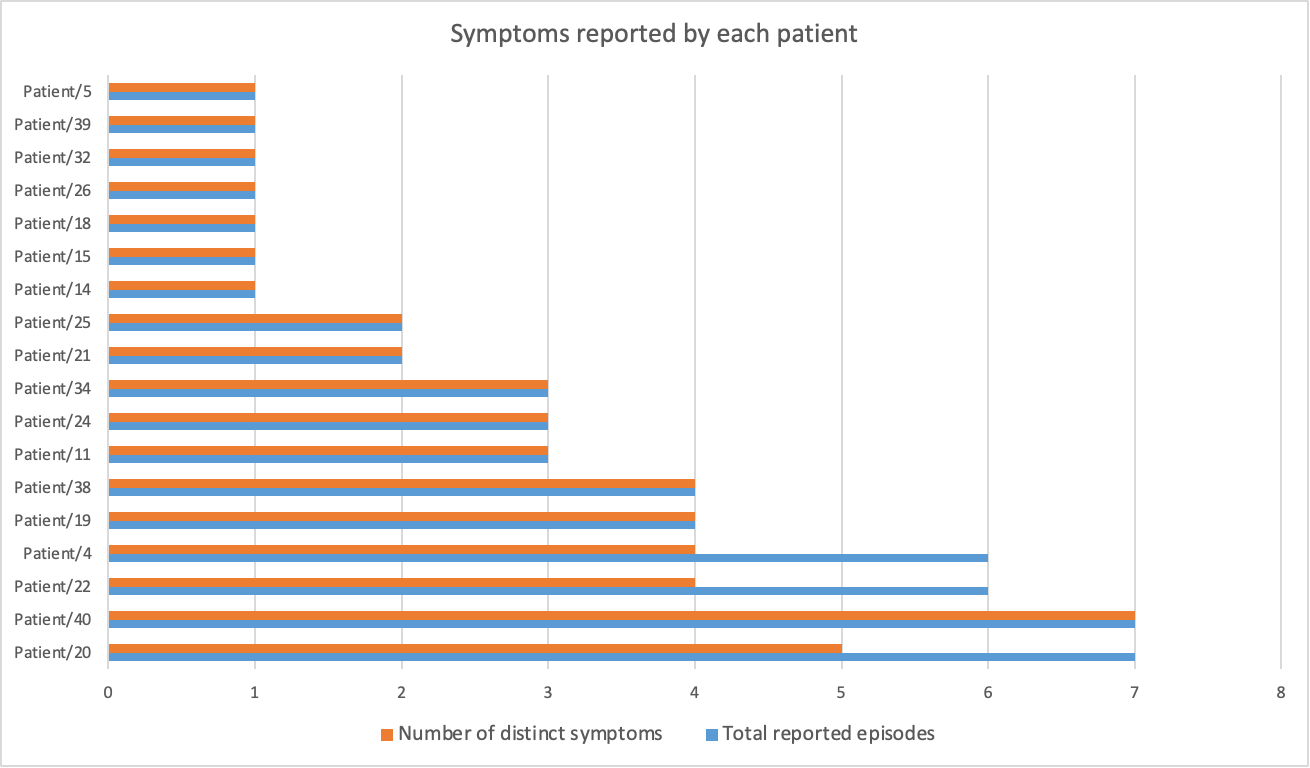
**

**Figure S2. Number of distinct patients who reported at least one symptom**

| **Table S5. Number of symptom episodes for each CTCAE grade for symptoms reported** | | | | | |
| --- | --- | --- | --- | --- | --- |
| **Symptom** | **CTCAE Grade** | | | | **Total** |
|  | **1** | **2** | **3** | **4** |  |
| Backache | 2 | 7 | 6 |  | 15 |
| Muscle pain | 2 | 6 | 5 | 1 | 14 |
| Fever | 5 | 3 |  |  | 8 |
| Joint pain | 5 | 2 |  |  | 7 |
| Limitation of joint movement | 1 | 2 | 1 |  | 4 |
| Diarrhea | 4 |  |  |  | 4 |
| Malaise | 1 |  | 2 | 1 | 4 |
| Pain in limb | 1 | 1 | 1 |  | 3 |
| Pain |  | 2 |  | 1 | 3 |
| Influenza-like illness | 2 | 1 |  |  | 3 |
| Cough | 2 |  |  |  | 2 |
| Fatigue | 1 | 1 |  |  | 2 |
| Headache |  |  | 1 |  | 1 |
| Xerostomia |  | 1 |  |  | 1 |
| Toothache | 1 |  |  |  | 1 |
| Difficulty walking | 1 |  |  |  | 1 |
| Pain in face |  | 1 |  |  | 1 |
| Mood anorexia |  |  | 1 |  | 1 |
| Stomatitis | 1 |  |  |  | 1 |
| Chill | 1 |  |  |  | 1 |
| Tremor |  | 1 |  |  | 1 |
| Nausea | 1 |  |  |  | 1 |
| Edema of face | 1 |  |  |  | 1 |
| Itching of skin | 1 |  |  |  | 1 |
| Total | 33 | 28 | 17 | 3 | 81 |

| **Table S6. Average symptom duration per closed symptom episode** | |
| --- | --- |
| **Symptom** | **Average duration (days)** |
| Fever | 11.6 |
| Joint pain | 35.7 |
| Diarrhea | 13.5 |
| Malaise | 33.2 |
| Cough | 5.1 |
| Fatigue | 15.1 |
| Influenza-like illness | 7.5 |
| Pain | 25.9 |
| Dry skin | 19.3 |
| Edema of face | 7.1 |
| Eruption | 27.8 |
| Headache | 1.5 |
| Hyperpigmentation of skin | 21.0 |
| Itching of skin | 2.0 |
| Limitation of joint movement | 68.7 |
| Muscle pain | 149.6 |
| Pain in face | 20.1 |
| Toothache | 7.2 |
| Tremor | 16.7 |
| Xerostomia | 5.8 |

| **Table S7. Engagement with well-being interventions** | | |
| --- | --- | --- |
| **Well-being intervention** | **N. of times executed** | **N. of patients** |
| My usual walk | 327 | 9 |
| Breathing exercise | 15 | 3 |
| Physical activity promotion | 11 | 1 |
| Yoga | 7 | 1 |
| Imagery training | 5 | 4 |
| Thai Chi | 0 | 0 |
